# Deck chair and reverse waist band sign in TIF 1Y positive dermatomyositis

**DOI:** 10.1016/j.ero.2025.09.005

**Published:** 2025-10-21

**Authors:** Sweta Subhadarshani, Leo Wang, Shivam Zaver, Kaylin Beiter

**Affiliations:** Department of Dermatology, University of Pennsylvania, Perelman School of Medicine, Philadelphia, PA, USA

A 49-year-old Caucasian man presented with a 6-month history of generalised pruritus and widespread erythematous rash occurring in the setting of elderberry supplementation for 1 month prior to symptom onset. His rash was initially treated as atopic dermatitis with failure of topical steroids and dupilumab.

By the time he presented to our clinic, he had progressed to erythroderma and had developed subsequent proximal muscle weakness, vocal hoarseness, and dysphagia. Cutaneous examination revealed near erythroderma with poikilodermatous and violaceous red scaly patches and plaques with sparing of folds and waist band area, gottrons’ and palmar papules, heliotrope rash, holster sign, nail fold telangiectasia, ragged cuticles, flagellate erythema, and poikilodermatous alopecic scaly patches on scalp. Mucosal examination was notable for a curdy white coating on his tongue, palate, and buccal mucosa (Figure 1). Muscular strength was reduced in proximal upper and lower extremities and hoarseness of voice was noted.

**Figure 1:**
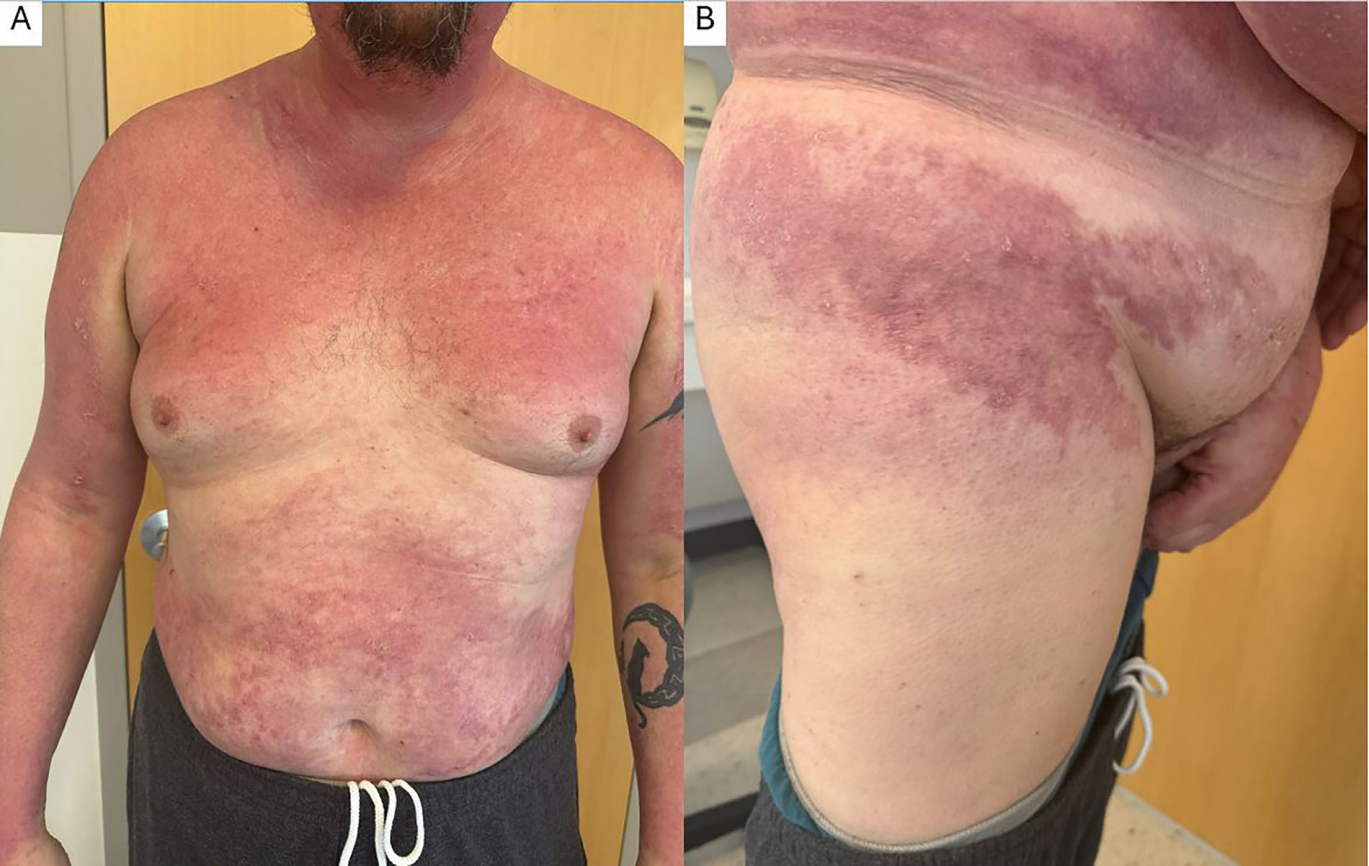
Patient image before treatment. (A, left) The patient’s physical examination findings from an anterior viewpoint. (B, right) The patient’s rash from a lateral viewpoint. Note the relative sparing of skin at body folds.

A biopsy was taken from lesional skin which demonstrated interface dermatitis with prominent mucin. Laboratory evaluation showed elevated Creatinine Kinase 919 (reference range: 49-397 U/L), aldolase 29 (reference range: 1-8 U/L), positive Anti-Nuclear Antibody 1:80 (reference range: <1:80) speckled without cytoplasmic staining, and a highly positive transcriptional intermediary factor 1 gamma (TIF-1Y) antibody (155 kDa) on an extended myositis panel (other myositis antibodies were negative including Sjogren's syndrome autoantibody A (SSA), Sjogren's syndrome autoantibody B (SSB), anti-ribonucleoprotein (RNP), anti-histidyl-tRNA synthetase (Jo-1), anti-alanyl-tRNA synthetase (PL-12), anti threonyl-tRNA synthetas (PL-7), antiaminoacyl-tRNA synthetase (EJ), anti-oligoclonal (OJ), anti-Signal Recognition Particle (SRP), Anti-Ku, PM/Scl-100, fibrillarin, Anti-Mi-2, Anti-Small ubiquitin like modifyer activating enzyme (SAE), Anti-Melanoma differentiation-associated protein 5 (MDA5), and NXP-2).

A diagnosis of TIF-1 positive dermatomyositis (DM) with candidiasis was made, and the patient was admitted and treated with high-dose steroids, intravenous immunoglobulin (IVIG), and fluconazole with subsequent improvement in muscle and skin symptoms. His candidiasis resolved with fluconazole, and no oesophageal infection was seen on esophagogastroduodenoscopy which was performed during this admission. A thorough paraneoplastic workup was negative during this admission. He was seen outpatient with initial improvement in his cutaneous and muscular symptoms, including being able to return to work, while treatment continued with monthly IVIG and steroids (Figure 2). Unfortunately, despite this initial improvement, he subsequently developed recurrent proximal muscular weakness and was readmitted for further management. Magnetic resonance imaging of the thigh demonstrated ongoing active myositis; his cutaneous symptoms did not significantly recur. Despite additional therapy with methotrexate, repeated steroids, and rituximab, he experienced a prolonged hospitalisation complicated by severe weakness and then ultimately passed away due to a gastrointestinal ulceration and perforation in the setting of chronic ileus.

**Figure 2:**
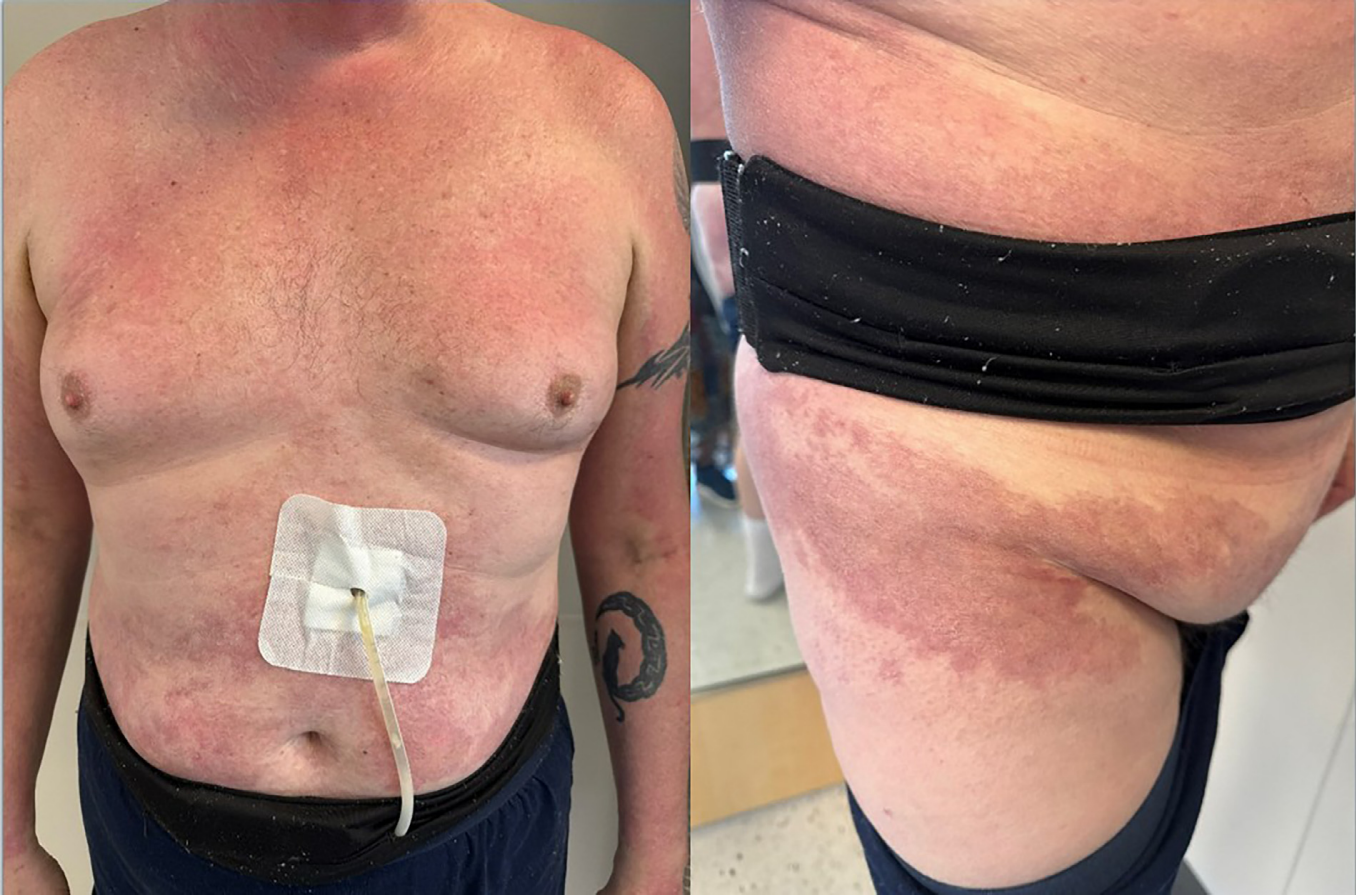
Patient image after treatment. (A, left) The patient’s physical examination findings from an anterior viewpoint. (B, right) The patient’s rash from a lateral viewpoint. While still present in both views, the patient’s rash demonstrates improvement that is most noticeable at the chest and abdomen. Note the patient’s gastrointestinal tube, which was required due to severe dysphagia which developed as part of his dermatomyositis.

Deck chair sign was originally described in papuloerythroderma of OFuji and refers to a phenomenon of the erythematous rash notably sparing natural skin folds (eg, axillary, inframammary, inguinal, and popliteal), thus resembling the slats of a deck chair. This has also been reported in Waldenstrom′s macroglobulinemia, adult T-cell leukaemia/lymphoma, angioimmunoblastic T-cell lymphoma, drug-induced erythroderma, and mycosis fungoides [1]. There are 4 prior reported cases (1 case series of 3 patients and 1 case report of this pattern in dermatomyositis) [2,3]. Three of the 4 patients were clinically amyopathic, of which 2 were positive for SS-A; 1 did not have a positive antibody. One patient had NXP-2 positivity and classic DM with rapidly progressing myositis with oesophageal involvement and passed away. There are no reports of this pattern of cutaneous involvement in a TIF-1Y positive myopathic dermatomyositis (Table 1). This may be an important diagnostic clue in erythrodermic patients with interface dermatitis without initial telltale signs of dermatomyositis. Furthermore, this patient in particular exhibited a reverse waistband sign, which refers to the sparing of areas covered by a natural waistband while involving the surrounding areas of the thighs and lower abdomen.

**Table 1:** Notable autoantibody clinical associations in dermatomyositis [5]

Antibody	Notable clinical associations
TIF1- γ	Malignancy; heliotrope rash; Gottron’s papules
NXP-2	Malignancy; severe calcinosis
MDA5 (CADM-140)	Mechanic’s hands; palmar papules; rapidly progressive interstitial lung disease
Mi-2	Cuticular overgrowth; shawl and heliotrope rash; flagellate erythema

The majority of physical examination findings in dermatomyositis are not specific to a specific autoantibody profile; the exceptions above are associations but not pathognomonic [5]. Clinicians should still perform autoantibody testing for definitive diagnosis. The most common associated malignancies in dermatomyositis include ovarian and gastrointestinal. Individuals should be followed closely with regular screenings for at least the first 3 years after diagnosis, the period during which cancer risk is highest. TIF: transcriptional intermediary factor 1 gamma; NXP2: nuclear matrix protein 2; MDA5: melanoma differentiation-associated protein 5; CADM-140: clinically amyopathic dermatomyositis antibody, 140 kd.

There is no literature on explanation of this pattern of involvement in dermatomyositis. We postulate that relative absence of stretch in intertriginous area may be at play given the role of stretching of dermal fibroblasts in upregulation of certain pathogenic molecules in dermatomyositis [4], [5].

## CRediT authorship contribution statement

**Sweta Subhadarshani:** Writing – review & editing, Writing – original draft, Supervision, Methodology, Investigation, Formal analysis, Data curation, Conceptualization. **Leo Wang:** Writing – review & editing, Writing – original draft, Data curation, Conceptualization. **Shivam Zaver:** Validation, Project administration, Investigation, Data curation. **Kaylin Beiter:** Writing – review & editing.

## Competing interests

All authors declare they have no competing interests.
